# Guidelines for measuring cardiac physiology in mice

**DOI:** 10.1152/ajpheart.00339.2017

**Published:** 2018-01-05

**Authors:** Merry L. Lindsey, Zamaneh Kassiri, Jitka A. I. Virag, Lisandra E. de Castro Brás, Marielle Scherrer-Crosbie

**Affiliations:** ^1^Department of Physiology and Biophysics, Mississippi Center for Heart Research, University of Mississippi Medical Center, Jackson, Mississippi; ^2^Research Service, G.V. (Sonny) Montgomery Veterans Affairs Medical Center, Jackson, Mississippi; ^3^Department of Physiology, Cardiovascular Research Centre, Mazankowski Alberta Heart Institute, University of Alberta, Edmonton, Alberta, Canada; ^4^Department of Physiology, Brody School of Medicine, East Carolina University, Greenville, North Carolina; ^5^Cardiac Ultrasound Laboratory, University of Pennsylvania, Philadelphia, Pennsylvania

**Keywords:** cardiac physiology, echocardiography, hemodynamics, magnetic resonance imaging, rigor and reproducibility

## Abstract

Cardiovascular disease is a leading cause of death, and translational research is needed to understand better mechanisms whereby the left ventricle responds to injury. Mouse models of heart disease have provided valuable insights into mechanisms that occur during cardiac aging and in response to a variety of pathologies. The assessment of cardiovascular physiological responses to injury or insult is an important and necessary component of this research. With increasing consideration for rigor and reproducibility, the goal of this guidelines review is to provide best-practice information regarding how to measure accurately cardiac physiology in animal models. In this article, we define guidelines for the measurement of cardiac physiology in mice, as the most commonly used animal model in cardiovascular research.

Listen to this article’s corresponding podcast at http://ajpheart.podbean.com/e/guidelines-for-measuring-cardiac-physiology-in-mice/.

## INTRODUCTION

The measurement of cardiac physiology is the foundation for assessing changes in anatomic and physiological features that occur within the myocardium during aging, in response to genetic alterations, and after a variety of experimentally induced pathologies. Cardiac physiology measurements also provide a means to examine the effects of therapeutic interventions.

With increasing concerns over data rigor and reproducibility, best-practice information is needed regarding measurements of cardiac anatomy and physiology in experimental settings. The focus of this review is to provide comprehensive guidelines on how to assess cardiac physiology in mice. We will discuss the importance of having a complete and rigorous physiological assessment when evaluating a number of cardiac pathologies. We will clarify what needs to be measured in different cardiac pathologies and discuss parameters to establish consistency both within and among laboratories. This guidelines article is a good companion for the article “Guidelines for animal models of myocardial ischemia and infarction” (106).

The sections in this article are divided by approach and include echocardiography, MRI, and hemodynamics measurements. Within each section, we discuss how each specific approach can be used to assess alterations in cardiac structure and function under different conditions, including studies of aging, cardiomyopathies such as postchemotherapy, diabetes, or sepsis, myocardial infarction (MI), and pressure overload cardiac hypertrophy induced by transverse aortic constriction (TAC), angiotensin II infusion, or isoproterenol infusion. Table 1 shows a list of suggested variables for assessment of cardiac physiology under these different conditions. The reference list serves as an additional resource to investigators new to the field.

**Table 1.** Suggested variables for assessing cardiac physiology under different conditions

Condition	Variables
Aging	• Dimensions, FS, volumes, EF, wall thickness • E and A waves (*E*/*A*; transmitral flow) • E′ and A′ waves (annular tissue movement) • To detect more subtle systolic changes, global LV systolic deformation (strain), or peak regional strain rate
Chemotherapy	• Dimensions, FS, volumes, EF, wall thickness • To detect more subtle systolic changes, global LV systolic deformation (strain), or peak regional strain rate
Diabetes	• Dimensions, FS, volumes, EF, wall thickness • To detect more subtle systolic changes, global LV systolic deformation (strain), or peak regional strain rate.
Myocardial infarction (MI)/ischemia-reperfusion (IR)	• Dimensions and FS (these indexes can be reported but are fraught with error in this model), volumes, EF (if possible, 3D reconstruction), wall thickness • Serial transverse LV sections to calculate the wall-motion score index • LV remodeling index, LV sphericity index • To detect more subtle systolic changes, global deformation and regional LV systolic deformation (strain), or strain rate • Left atrial size • *E*/*A*, E′ and A′ waves
Hypertrophy and dilated cardiomyopathy (with or without hypertrophy)	• Dimensions, FS, volumes, EF, wall thickness • LV hypertrophy index • *E*/*A*, E′ and A′ waves • Deceleration time, isovolumic relaxation time • Left atrial size • To detect more subtle systolic changes, global LV systolic deformation (strain), or peak regional strain rate

FS, fractional shortening; EF, ejection fraction; LV, left ventricular; *E*/*A*, E wave-to-A wave ratio; EDD, end-diastolic dimension; EDV, end-diastolic volume; ESD, end-systolic dimension; ESV, end-systolic volume. Dimension-based calculations are as follows: EF = [(EDV − ESV)/EDV] × 100, FS = [(EDD − ESD)/EDD] × 100, LV hypertrophy index = EDD/wall thickness (at diastole), LV remodeling index = EDV/LV mass, and LV sphericity index = EDV/volume of a sphere with a diameter equal to EDD.

## ECHOCARDIOGRAPHY

Echocardiographic imaging is a widely used, noninvasive means to assess cardiac physiology and architecture in rodent models of heart disease and aging and allows for repeated assessment of heart function over the course of disease progression (8, 15–18, 35, 41, 50, 61, 74, 79, 84, 86, 88, 91, 112, 113, 123, 125, 126, 136, 142, 143, 150–153, 163, 164, 168, 171, 175, 177, 191, 202, 205, 212–214). One reason for its appeal is that echocardiographic ultrasound imaging provides a comprehensive array of information on cardiac anatomy, physiology, and mechanical properties. While ultrasound can also be used to obtain information from the vasculature (arteries and veins), we will focus on the application of echocardiography in analyzing cardiac structure and function. To obtain reliable and reproducible information from echocardiography that can be compared across laboratories, a number of factors need to be considered. These criteria are outlined in terms of the type and depth of anesthesia, the mode of recording, and the (space-time) variables most informative for accurate and precise assessment of various models of heart disease and related interventions.

Echocardiographic imaging uses high-frequency sound beams that penetrate the thoracic cavity and are reflected back to the ultrasound transducer when they reach an interface among moieties of different acoustic impedance, such as the myocardium, blood, valves, or vessel wall. This reverberated signal is then processed by the instrument software to produce a real-time image of the heart (or vessel). Echocardiography uses four main principal imaging formats: two-dimensional (2-D) brightness mode (B-mode), motion mode (M-mode), Doppler imaging, and three-dimensional (3-D) imaging. Unique challenges facing echocardiography in mice include the small heart size (5- to 8-mm length in an adult mouse) coupled with high heart rates (400–650 beats/min in an unanesthetized mouse, depending on the strain). Recent technological developments include high-frequency transducers (up to 70 MHz) and enhanced imaging frame rates to provide high spatial and temporal resolutions to view structural and physiological changes in the left ventricle (LV). In addition, integration of respiration, heart rate, and ECG monitoring during ultrasound recordings allows for additional quality control (and potential normalization) during recording. Echocardiography is now commonly used to measure different aspects of cardiac architecture (wall thickness and chamber dilation) and physiology (systolic and diastolic) in rodents.

Cardiac hypertrophy and dilated cardiomyopathy are generally associated with a uniform structural remodeling of the LV, and, as such, M-mode and B-mode imaging are suitable approaches to measure LV wall thickness (septal and free wall) and LV chamber size. MI (permanent occlusion of the left anterior descending artery), on the other hand, is associated with nonuniform remodeling of the LV chamber due to the scar (infarct) formation that replaces a major fraction of the LV free wall (and sometimes the septum). In this scenario, M-mode imaging is limited in its assessment capacity; global changes in the structure of an infarcted, remodeled LV are better assessed using 2-D or even 3-D images. ECG-gated kilohertz visualization (EKV) is a 3-D reconstruction of the heart using imaging gated to the ECG (20). With EKV, one can obtain serial M-mode images of the LV (from the apex to base), which are then spatially and temporally reconstructed into an ultrasound B-mode image data set for one cardiac cycle. The high temporal resolution allows for better tracking of myocardial borders and can be used to measure LV chamber size and LV contractility (121).

### Anesthesia

The first step in preparing for echocardiographic imaging is to decide what, if any, anesthetic to use. Some studies have reported that ultrasound recordings can be performed on conscious, carefully restrained mice to avoid an anesthesia-induced decrease in heart rate that can influence cardiac function (203). Mice can be imaged free hand or restrained on a platform using elastic cord or tape. In the latter setting, ECG electrodes would be taped to the paws (46). It is important to note that the acquirement of reliable results from ultrasound recordings in conscious animals requires that the mouse be sufficiently acclimatized to the imaging environment (e.g., the platform, restraining devices, use of warm gel) and to the individual capturing the images. This will significantly influence the demeanor of the animal, as attempts should be made to avoid a stress-related rise in heart rate that can subsequently have an impact on cardiac performance. While the two schools of thought (anesthetized vs. awake) continue to exist and evolve, it is critical to recognize that either approach should be done under conditions that minimize procedure-related changes to cardiac function and that echocardiography data acquired from anesthetized mice should not be compared with those obtained from conscious mice.

The anesthetics that have been most commonly used for echocardiography in mice are isoflurane and ketamine-xylazine, while other anesthetics, such as tribromoethanol (Avertin), medetomidine, pentobarbital sodium, and ketamine, in combination with other anesthetics (including midazolam and fentanyl), have also been occasionally used in mice (48, 90). Ketamine alone and avertin have both been shown to keep heart rates in the range of 550−beats/min and have little cardiodepressant effects (195, 212).

A mixture of ketamine (an *N*-methyl-d-aspartate receptor antagonist, 100 mg/kg) and xylazine (an α_2_-adrenoceptor agonist, 10 mg/kg) has been previously and prevalently used as the anesthetic of choice for surgeries as well as for echocardiographic imaging. However, in the past two decades, numerous studies have demonstrated cardiodepressant effects of ketamine-xylazine, which have made this choice of anesthetic unacceptable for physiological measurements. This is mainly due to the cardiodepressant effects of xylazine, which can significantly reduce heart rate and LV function compared with isoflurane (135, 146). Isoflurane (1–2%) has become the most popular anesthetic for echocardiography in mice (78, 86, 88, 107). As the hemodynamic effects of anesthesia vary over time, care must be taken to acquire echocardiograms at the same time after induction and at a similar heart rate. For example, a 10-min wait period after the mouse is placed on the board provides a uniform acclimation period. A heart rate of >400 beats/min is advised to be within the physiological range of murine heart rate under anesthesia. In a recent study (135), the effects of different anesthetics on LV systolic function were evaluated. While it is important to sustain a high heart rate during assessment of cardiac function in rodents, heart rates of >650 beats/min suggest activation of the autonomic nervous system, and, therefore, results with heart rates of <400 and >650 beats/min should be interpreted with caution (116, 199). A reduction in the heart rate (<400 beats/min), secondary to the type or depth of anesthesia, suppresses LV systolic and diastolic function, whereas an excess rise in heart rate (>650 beats/min, e.g., due to stress or activation of the autonomic nervous system) can result in insufficient LV filling. As discussed later, heart rates of >500–600 beats/min are also accompanied with fusion of LV filling waves [early (E wave) and atrial (late, A wave)] and the inability to assess diastolic function in mice.

In choosing an anesthetic for echocardiography, additional criteria, beyond heart rate and LV systolic function, need to be considered. Table 2 shows a list of common anesthetics used for echocardiography in mice, with reported advantages and limitations. Some anesthetics have been reported to have protective effects on cardiac recovery from surgery under certain dosage and duration conditions (49, 109, 183). Important factors in choosing an anesthetic include ease of handling, amount of stress induced, ability to adjust level and duration, impact on physiological parameters (blood pressure, heart rate, and cardiac function), and recovery time. Moreover, while the decision of choosing an anesthetic (or none at all) is often based on the effects on heart rate, it is important to note that if cardiac diastolic function is also to be assessed by echocardiography, at very high heart rates, then the E and A waves (or the tissue Doppler equivalents, E′ and A′ waves) can become fused (merged), therefore preventing accurate measurement of diastolic function. A rapid assessment using free-hand scanning can be done in <5 min, while for a more thorough echocardiogram acquired over 10–20 min, stable anesthetic conditions are advised.

**Table 2.** Anesthetics commonly used to sedate mice to acquire physiological measurements (2, 28, 55)

Anesthetic	Dosage range	Advantages	Limitations
Isoflurane	1–3% (1 l/min)	• Fast acting • Short lasting	• Cardiorespiratory depression
Barbiturates	30–90 mg/kg	• Short or long acting	• Cardiorespiratory depression • Hypotension
Ketamine	80–100 mg/kg	• Less respiratory depression • Preserves cardiovascular physiology	• Very light sedation • Can induce seizures
Ketamine/xylazine	80–100 mg/kg; 5–15 mg/kg	• Can be combined with opioids and analgesics	• Bradycardic and hypotensive
2,2,2-Tribromoethanol (avertin) or 2-methyl-2-butanol	240 mg/kg	• Not a controlled substance • Short acting • Moderate • Cardiopulmonary depression	• Peritonitis, intestinal ileus • Abdominal adhesions • Light sensitive (toxic byproducts)

### B-Mode Imaging

B-mode produces 2-D views of the heart (short or long axis) and allows for assessment of cardiac chamber dimensions, cardiac physiology, and visualization of cardiac anatomic structures, such as papillary muscle and valves. In cine loops obtained from B-mode, the LV endocardium can be traced in diastole and systole to calculate cardiac ejection fraction (EF). Similarly, LV mass can be estimated by tracing the epicardium and endocardium in a midventricular parasternal short-axis view. B-mode also serves as an orientation guideline for regions of the heart that require further assessment by other imaging modes, such as M-mode or color Doppler imaging. Imaging of the right ventricle (RV) in the short-axis view in the mouse is challenging, due to interference from the sternum not allowing proper positioning of the probe for RV imaging; detailed protocols have been developed for using echocardiography to assess RV structure and physiology (25, 96, 122).

Among the currently available imaging modes, EKV provides maximum frame rate imaging with maximum resolution, allowing for increased temporal and spatial resolution imaging (20, 121). As a comparison, with B-mode imaging, acquisition occurs at frame rates of 300 frames/s compared with ~1,000 frames/s with EKV recording. This mode of imaging can be useful in assessing cardiac structure and physiology, where the LV undergoes wall thinning (e.g., in MI) when high-resolution images are required to visualize the infarcted thin and dyskinetic LV wall (87, 171).

### M-Mode Imaging

M-mode images are obtained by a rapid succession of B-mode scans along a single axis displayed over time. M-mode images appear as a continuous tracing, showing the motion of the myocardial walls as they contract during systole and relax during diastole. This mode provides a very high temporal resolution of LV wall motion to assess LV contractile patterns and chamber size. Parameters that can be obtained from M-mode imaging include LV internal diameter (LVID) at end systole (LVIDs) and end diastole (LVIDd), which can then be used to calculate fractional shortening (FS) as a measure of systolic function and cardiac contractility [FS = (LVIDd − LVIDs)/LVIDd × 100]. M-mode images can also be used to derive additional functional parameters such as calculated EF, cardiac output (CO), and stroke volume (SV) or structural parameters such as LV mass. Calculations of LV volumes and EF from M-mode, however, have a number of geometric assumptions that must be taken into account. In calculations derived from M-mode dimensions, the LV is assumed to be of a spherical (symmetric) rather than an oval (asymmetric) shape. This is not accurate and leads to even greater errors in pathological cases, such as post-MI remodeling. Similarly, while the calculated LV mass is often comparable with actual mass, the gravimetric measurement of LV mass remains the gold standard.

### Doppler Imaging

This imaging mode uses the Doppler shift principle, reflected by the moving target, to determine blood flow velocity and direction, as evidenced by color differential. In the case of color Doppler, the moving target is blood cells (moving parallel to the beam); in the case of tissue Doppler, the moving target is the myocardium. An increase in blood flow will be reflected as an increase in Doppler shift. In pulsed-wave Doppler, the transducer transmits and receives the sound waves, whereby the blood flow-velocity profile is determined from a precise location (determined by 2-D image guidance). Pulsed-wave Doppler can be used to measure transvalvular flow-velocity profiles, which are particularly useful in assessing cardiac diastolic function, including isovolumic relaxation time (IVRT), E wave and A wave ventricular filling velocities, and deceleration of the E wave. More details on accurate assessment of diastolic function are provided in later sections. Pulsed-wave Doppler can also measure systolic parameters, such as ejection time (ET), isovolumic contraction time (IVCT), and IVRT. These parameters provide information on the kinetics of LV systole (ET and IVCT) and cardiac diastole (IVRT). For instance, prolonged ET or IVCT may reflect a reduced rate of contraction, whereas an increase in IVRT often reflects impaired diastolic function.

Color Doppler imaging uses a color-encoded map of flow velocity and direction superimposed on the 2-D image. Blood flowing toward the ultrasound transducer is identified in red (increase in frequency), blood flowing away from the transducer is depicted in blue (decrease in frequency), and blood flowing horizontally is not detected. Doppler evaluation of blood flow and velocity can also be obtained in any vascular bed. One of the possible applications of Doppler imaging to measure blood flow and velocity is in determining the severity of pressure overload in the TAC surgical model to induce cardiac hypertrophy (88, 136). Presence of a turbulent flow results in a mosaic of colors at the site of constriction, where pressure and flow gradient can be obtained through pulsed-wave Doppler. Pulsed-wave Doppler can also be used to measure the pressure gradient, which can be particularly useful in TAC to ensure that all mice in a study are subjected to the same degree of aortic constriction. It must be noted, however, that a limitation of pulsed Doppler is that it cannot measure high velocities; the maximum pressure gradient that can be measured is ~60 mmHg.

Tissue Doppler imaging is used to assess global and regional cardiac function. Mitral annulus measurements of motion velocity are used to assess E′, A′, and S waves (LV diastolic parameters of relaxation). Myocardial velocities can also be measured and strain (deformation) rates derived from these measures. Peak systolic myocardial strain rate is a relatively load-independent measure that can identify subtle changes in systolic function. One limitation of the Tissue Doppler imaging method is that it only measures velocities parallel to the beam; therefore, analyses of radial function are limited to the anterior and posterior walls, and the circumferential function is limited to the septal and lateral walls.

### 3-D Images

M-mode and B-mode images represent a grayscale of amplitude in one and two dimensions. 3-D echocardiography in mice is based on reconstruction from multiple 2-D images. The images can be digitally stacked as a series of short- or long-axis views. Alternatively, a semi-automated 3-D acquisition image, obtained from varying transducer positions at the same points in the cardiac cycle (gating), measures LV chamber volume during end systole (LVESv) and end diastole (LVEDv), which are used to calculate EF [EF = (LVEDv − LVESv)/LVEDv × 100]. Whereas 2-D echocardiography is based on the symmetric LV shape and structure assumption, 3-D imaging avoids LV shape assumptions, a particularly useful feature in assessing LV dilation and dysfunction in a model of MI (40). It is important to emphasize that EF, whether in 2-D or 3-D space, is highly dependent on loading conditions (preload or afterload).

### Speckle-Tracking Imaging

Speckle tracking is a novel, non-Doppler-based technique used to detect myocardial displacement, wall motion velocity, and myocardial deformation indexes, such as strain (fractional change in length of myocardial segment) and strain rate (the rate of change in strain). Strain and strain rate detect early LV dysfunction before changes in EF occur (7). Similar to EF, strain is also load dependent, whereas strain rate appears to be less load dependent. In speckle-tracking imaging, LV endocardial and epicardial borders are traced to form a region of interest, and speckle patterns are identified inside this region of interest. The group of speckles that correspond to each region of the myocardium can be tracked from frame to frame using a speckle-tracking algorithm. The resulting geometric shift during a cardiac cycle is used to calculate displacement, regional velocity (displacement per unit time), strain, and strain rate along the radial, circumferential, and longitudinal planes of the heart. The advantage of speckle tracking over Doppler imaging is that quantitative speckle-tracking assessment of myocardial performance is not angle dependent. The frame rate used to measure strain rate needs to be high (at least 350–500 frames/s depending on heart rate) to capture the maximum strain rate, which is a brief event.

Displacement can be measured along the radial and longitudinal axes as the distance traveled by the speckles from peak systole to peak diastole. In addition, strain and strain rate can be used to assess regional myocardial function. Radial strain (percent change in myocardial wall thickness) can be measured along the short or the long axis and is depicted as a positive curve during systole (reflecting increasing wall thickness) and a negative curve during diastole (reflecting decreasing LV wall thickness). Circumferential strain is obtained from a short-axis view and represents the percent change in myocardial circumference, whereas longitudinal strain detects the percent change in LV length (from the apex to base). Time to peak analysis is another index measured by speckle tracking that is used to assess dyssynchronous contraction among different myocardial regions (21). In this analysis, the LV myocardium (short- or long-axis image) is divided into six segments. In a healthy LV, all segments should be synchronized (similar velocities), peaking at similar times. Under pathological conditions, certain segments may move at a different velocity (e.g., due to the presence of fibrotic lesions) and therefore peak at different times, resulting in dyssynchrony. Peak regional strain values can be reported for specific LV segments, whereas averaging peak strain and strain rate measurements across all six segments can be reported as global strain (19, 144).

Regional strain per LV segment (rather than global strain) can provide useful information in a post-MI model, since there is a clear regional difference in LV structural remodeling and function. As such, summation of all segments could mask regional differences. In hypertrophic or dilated cardiomyopathy associated with focal fibrosis, enhanced stiffness of a fibrotic segment reduces regional velocity, resulting in dyssynchrony. Therefore, speckle-track imaging can provide very useful information regarding the alterations within the myocardium that may be too subtle to be detected by conventional imaging modes. One important caveat with speckle tracking is that different ultrasound machines use their own proprietary software to acquire and track the speckles, resulting in a lack of standardization of strain and strain rate values. Care must be taken to acquire serial echocardiograms on the same equipment.

### Assessment of LV Systolic Physiology

Systole is the contraction phase of the heart that ensures normal ejection of blood from the chambers into the arteries to supply the body with oxygen. The most commonly used variables to evaluate systolic function are FS and EF, both provided as percentage values during systole compared with diastole. FS indicates the percentage change in LV chamber size and is an index of myocyte contraction, whereas EF indicates the percentage change in LV volumes and is an index of LV function. Other systolic physiology variables, such as SV and CO, can be calculated from measurements of LV wall thickness and chamber dimensions. The volumes and EF derived from M-mode are generally calculated using the cubed formula (diameter^3^), which assumes the heart is a sphere. Volumes and EF derived from 2-D measurements assume a symmetrical LV geometry, which, of course, will not be the case in cardiac pathologies, such as MI. FS and EF are calculated based on LV chamber diameter and volume. Heart rate positively correlates with FS and EF, and, therefore, a drop (or rise) in heart rate, due to anesthetic, can lead to altered systolic function assessment. For instance, low heart rate associated with deep anesthesia is accompanied with LV dilation and overall cardiac depression. Therefore, the maintenance of a stable heart rate throughout the echocardiographic recording is particularly important, especially when assessing cardiac function in postinjury hearts that exhibit LV remodeling.

After pressure overload, LV volume first decreases, due to thickening of the LV wall (compensatory hypertrophy), followed by LV dilation and enlarged LV volume. Since the LV undergoes a uniform remodeling (LV wall thickness is altered similarly in the free wall and the septum), M-mode imaging can be used to measure LV chamber diameter, from which FS can be calculated as FS = [(LVIDd − LVIDs)/LVIDd × 100].

After MI or ischemia-reperfusion (I/R), there is nonuniform LV remodeling that includes thinning of the LV free wall and thickening of the septal nonischemic LV wall. Therefore, M-mode imaging in these cases provides an incomplete and sometimes false evaluation of LV remodeling and function. Additionally, M-mode could provide a biased view of the LV if the probe is not placed in the same location across all of the mice examined, particularly across control and MI groups. Therefore, it is our recommendation that M-mode measurements not be used as end points in this model. We recommend that images are acquired and analyzed according to American Society of Echocardiography guidelines for humans, tracing from leading edge to leading edge or trailing edge to trailing edge. While 2-D imaging provides clear views of wall motion abnormalities, several planes need to be imaged to delineate the extent and geometry of the abnormality. For this reason, small wall motion abnormalities can be missed. To circumvent this issue, 3-D imaging to evaluate LV volume (rather than LVID) and to calculate EF [EF = (LVEDv − LVESv)/LVEDv × 100] is superior and more accurate compared with FS measurements. LV volumes can be measured from several short-axis views using the Simpsons method (153), which represents the LV cavity as a stack of disks, and LV volume is calculated as a summation of all disks.

LV function post-MI or post-I/R can also be assessed by determination of the wall motion score index (WMSI) (207), which is calculated based on a 10- to 16-segment model on short-axis views and is scored as 1 for normal, 2 for hypokinetic, 3 for akinetic, 4 for dyskinetic, and 5 for aneurysmal. WMSI is calculated as the sum of scores divided by the total number of segments that were analyzed. A higher WMSI value corresponds to a greater degree of LV dysfunction. This parameter, along with EF, provides information on LV systolic function post-MI or post-IR (86, 145, 171). As EKV yields a higher temporal and spatial resolution compared with B-mode imaging, the use of EKV recording to assess LV volumes and WMSI in post-MI hearts can provide a more accurate assessment of these parameters.

As in humans, an injection of contrast microbubbles can improve the definition of the endocardium. Additionally, the RV can be visualized. This approach, however, necessitates an intravenous injection. Care must be taken to inject a small volume of fluid (<20 µl) so that hemodynamic conditions are not disrupted by the injection.

The Tei index or myocardial performance index provides an overall, combined assessment of both the systolic and diastolic function of the heart: (IVCT + IVRT)/ET. While the Tei index was originally and erroneously used to report diastolic dysfunction, it is an index of cardiac performance during a complete cardiac cycle (systole and diastole) (173). It has been previously reported in mice but is not widely used, because the Tei index does not correctly reflect changes in heart function in some disease models (159).

### LV Diastolic Physiology

Diastole is the relaxation phase of the heart that ensures normal filling of the ventricles during rest and provides adequate blood volume to maintain normal CO. Diastolic dysfunction is defined as an impairment in active relaxation, passive stiffness, or the combination. Assessment of diastolic dysfunction in animal models of heart disease has received significantly more attention, since heart failure with preserved EF has become increasingly recognized in patients (133, 154). Assessment of LV diastolic function in mice includes evaluation of LV filling velocity measured by the magnitude of E and A waves as well as E′ and A′ waves (tissue Doppler imaging), IVRT, E wave deceleration time (DT), and left atrial (LA) size. These parameters have been used to assess reliably diastolic dysfunction in mice (17, 123, 171).

It is important to note that the size of the mouse is a critical factor in selecting appropriate parameters to assess diastolic dysfunction accurately. A word of caution: a number of parameters, such as pulmonary venous flow and transmitral flow propagation velocity, which have been proven to be useful in patients, cannot be used in rodent models, due to the small size of the heart and fast heart rate in these animals, which restrict the spatial and temporal resolution beyond the detection limits of currently available technologies. Furthermore, the variability of these indexes among individual mice contributes to the difficulty and inaccuracy/inconsistency of diastolic assessment in this species.

### Mitral Flow Profile

The mitral flow profile comprises two waveforms, the early filling (E wave) and late or atrial filling (A wave), and provides information about the LV filling dynamics (155). The E wave represents the blood flow through the mitral valve during the early filling phase of the LV and can be affected by the rate of relaxation and compliance of the LV. The A wave represents the blood flow (through the valve) during the atrial contraction phase and can be altered by LA contractility or compliance (100). The E wave to A wave ratio (*E*/*A*) can be used as a measurement of diastolic dysfunction (if *E*/*A* < 1), however, not in isolation; as outlined by the new diastolic guidelines from the American Society of Echocardiography, *E* and *A* must be analyzed in the context of myocardial disease, annular tissue velocities, and LA size (127).

Mitral flow profile also provides information on DT and IVRT. DT is the time required for pressure equilibration between LV and LA and is measured as the time from the peak of the E wave to the baseline. IVRT is the time from closure of the aortic valve to the opening of the mitral valve. Increased IVRT represents prolonged LV relaxation. Although these measurements provide valuable information on LV filling kinetics, these parameters can be influenced by a number of factors, such as preload, arrhythmia, or very high heart rate as well as diseases that cause a hyperdynamic state. Furthermore, normal values of DT in mice are not well reported, and IVRT−a very short time interval−can be difficult to measure.

### LA Size

LA size can be used as a marker for chronic elevation of LV filling pressure and is often used to assess diastolic dysfunction in humans (83, 139) and animal models (17, 50, 123, 171). Given the small LA size in mice, strict attention, with respect to probe location, is required for accurate measurements. M-mode, in the parasternal long-axis view, can be used to measure the maximal anteroposterior LA diameter in rodents (17). We recommend normalization of the LA diameter by the tibial length to avoid variability due to individual animal size. Other indexes, such as the use of pulmonary venous flow and transmitral flow propagation velocity, have been reported to correlate with elevated LV filling pressures in mice but are not routinely used (33, 68, 179).

### Other Specific Echocardiographic Techniques

Assessment of cardiac physiology by echocardiography would not be complete without mentioning the possibility to assess ischemia and coronary reserve using stress echocardiography, Doppler-derived coronary reserve and myocardial perfusion using contrast echocardiography (5, 62, 142, 143, 190). Additionally, in pulmonary and RV pathologies, the measurement of pulmonary acceleration time, which has been used extensively in humans as a measure of pulmonary pressure, has been validated in mice (175). On the Vevo 3100, there is a noncontrast power Doppler mode, which provides information on vascularity that has mostly been used for oncology or muscle studies and color Doppler. It is possible for vessels to be studied using high-frequency probes, yielding information on their size and distensibility, thus providing additional information on the ventricular–arterial coupling. In our experience, the power Doppler mode has been difficult to interpret in the heart.

### Literature Analysis of Articles Published in American Journal of Physiology Journals Using Echocardiography

To evaluate the current reporting of echocardiographic results, we accessed PubMed on July 21, 2017, and searched for “mouse and *American Journal of Physiology* and (echocardiography or echocardiographic or ultrasound).” This search resulted in 437 articles, of which 52 articles were published between January 1, 2016, and present as well as 14 articles that were excluded as false positives (two kidney, one liver, one lung, one bone, one RV, two rat not mouse, one fetal not adult, and five with no cardiac echocardiography results). The remaining 38 articles were analyzed both for completeness of information provided and for quality assessment of the echocardiography values reported (11, 13, 22, 27, 30, 36, 38, 39, 42, 45, 52, 54, 57, 59, 65, 70, 75, 76, 78, 85, 102, 114, 117–119, 132, 147, 148, 158, 160, 165, 170, 172, 182, 187, 193, 201, 206).

**Fig. 1.**
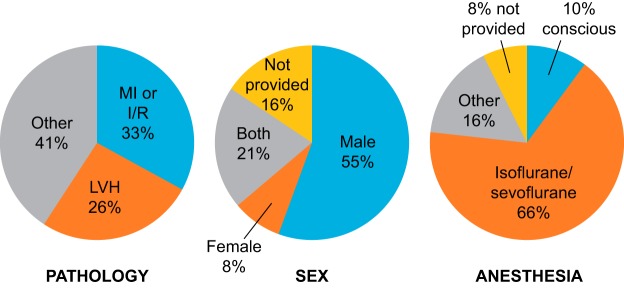
Results of a literature analysis of articles published in the *American Journal of Physiology* since January 1, 2016. The pie charts show the percentages of articles that covered different pathologies (*left*), divided by the sex of the mice (*middle*), and separated by the anesthesia used (*right*). LV hypertrophy (LVH) included genetic models as well as angiotensin II infusion or transverse aortic constriction models of pressure overload.

A summary of the pathologies examined, mouse characteristics, anesthesia used, and results reported is shown in Fig. 1. The pathology assessed was approximately equally split between MI and LV hypertrophy models. Over one-half of the studies used male animals only; of note, 21% of the studies used both sexes. Isoflurane was the most common anesthetic used, with very few studies using conscious animals. A total of 68% of the articles used the C57 strain, although this was inconsistently provided as C57, C57BL/6, or C57BL/6J; while it is assumed all of these mean C57BL/6J, only 8 of 26 articles (30.8%) in this category specified the exact strain details. There was an even split in how results were presented, with 44% using tables and 56% using graphs. Most reported statistical analysis details, although not all specified which test was used for each evaluation. A surprising 58% did not report heart rates, and 82% did not report details on whether acquisition and analysis were blinded. While 92% provided some details on what instrument and probe were used, what views were acquired, and how the probe was positioned, very few provided sufficient details to assess completely quality of acquisition or analysis.

There was a wide range of values reported for each parameter, even for healthy control mice; this is likely due, in part, to the wide range of conditions under which imaging was performed. A total of six studies in healthy wild-type mice, using similar conditions (C57BL/6J, one of the Vevo instruments, isoflurane), provided results in tables so that we could obtain mean values and had results that the committee considered to be of high quality. These results were combined and are shown in Table 3. Of note, awake mice or mice anesthetized using different anesthetic regimens (e.g., ketamine alone) would be expected to have smaller LV dimensions and higher FS and EF. Based on this analysis, we developed a checklist for authors and reviewers on the minimum information that should be provided when reporting echocardiographic experiments (Table 4).

**Table 3.** Compilation of echocardiography results in healthy male C57BL/6J mice from 6 studies using similar instrument and anesthesia protocols (13, 45, 78, 117, 170, 201)

	Left Ventricular Internal Diameter at End Diastole, mm	Left Ventricular Internal Diameter at End Systole, mm	Fractional Shortening, %	Ejection Fraction, %
Number of mice	5	6	6	3
Low	2.8	1.7	34	60
High	3.8	2.6	45	75
Mean	3.4	2.1	40	69
SD	0.4	0.3	4.1	8.2
Coefficient of variation	12	15	10	12

**Table 4.** Checklist for authors and reviewers: minimum details needed for cardiac physiology methods and results

Methods: • Mice: strain, age, sex • Instrument used: model, probe type and placement, views acquired • Anesthesia: type and amount • Analysis: measured versus calculated measurements • Ejection fraction: formula used • Blinding for acquisition or analysis (both are recommended) • Statistical analyses used
Results: • Report heart rates • If showing normalized values, provide raw values at baseline • Tables or graphs: recommend reporting results in tables; if using graphs, include mean values of main parameters in text

## MAGNETIC RESONANCE IMAGING

MRI is a noninvasive, high-resolution imaging technique that can be used to assess myocardial anatomy, perfusion, wall motion and contractility, and physiology in mice (58, 192). Myocardial molecular imaging/tagging is not discussed here to maintain the focus of this discussion on cardiac physiological measurements; please refer to several excellent articles regarding its use (140, 161, 185, 189, 198). The use of MRI in mice has two major challenges: the small size of the heart (spatial resolution) and elevated heart rates (typically 400–650 beats/min depending on the strain) that can introduce motion artifacts. These challenges, in addition to respiration and signal-to-noise ratio limitations, can severely deteriorate image quality. While MRI is the gold standard in the clinic, its use in mice is still in development. Unlike human imaging, which can use single slice acquisitions, imaging of the mouse heart requires stacking images obtained at a particular time in the cardiac cycle to generate the slice image (26). Therefore, in thinned tissue, the imaging quality may not be as robust. Tailored imaging protocols and dedicated cardiac hardware and software are essential to achieve appropriate temporal and spatial resolution for cardiac MRI in rodents. Below, we provide guidelines for cardiac MRI in mice.

### Hardware

Dynamic imaging of the myocardium requires high ventricular blood-to-myocardium contrast, full coverage of the cardiac cycle, and high temporal and spatial resolution. For this reason, gradient echocardiography-based imaging techniques are used (97). MRI systems for mice have small bore magnets imaging at very high field strengths (>7 T) (188). Increasing field strength for MRI increases the signal-to-noise ratio, resulting in higher resolution. The increase of field strength has the trade offs of also increased lack of field homogeneity, susceptibility to artifacts, and higher radiofrequency power deposition (124). While the full range of field strengths uses a span from 1.5 to 3 T, using optimized coils at the low end (58, 67) to 17.6 T at the high end (66), the range of 7–11.7 T offers the best compromise between resolution and presence of artifacts for mice imaging (47).

### Gating Strategies

To limit motion artifacts, it is essential to perform cardiac gating to acquire quality images. Images should be acquired over several cardiac cycles and simultaneously with an ECG for synchronization, a method commonly designated as prospective gating (54). Prospective gating involves MRI acquisition at a predefined portion of the cardiac cycle (for example, at diastole) after detection of the upslope of an R wave in the ECG (97). While there are some variations in mouse MRI protocols across laboratories, basic principles are commonly followed (shown in Table 5). First, with few exceptions, mice are under anesthesia and allowed to breathe freely. Isoflurane inhalation is currently the preferred approach, due to quick anesthesia induction and fast awakening, minimal hemodynamic depression, and easy regulation of anesthesia depth (98). Mice usually receive 1.0–2.0% isoflurane, 30–50% oxygen, and 50–70% air (69). Second, temperature (by rectal probe), heart rate (400–650 beats/min, depending on the strain), and breathing activity (~50–100 cycles/min) are closely monitored and controlled (29). Third, the animal’s position has to be reproducible; this is a critical step and can affect data quality and the degree to which motion artifacts affect the imaging. The animal is placed in a prone position (fixed with tape or plastic pins to an animal sleigh or other fixation device), with the anesthetic agent supplied through a nose cone, the breathing sensor normally attached around the abdomen, and the temperature measured by a fixed rectal probe. Fourth, prospective gating synchronized to the ECG has to be performed. Finally, a multiphase gradient echocardiography-based cine cardiac imaging is acquired and the data are analyzed.

**Table 5.** Basic principles followed during MRI in mice

Anesthesia	• Isoflurane at 1–2% • O_2_ at 30–50% • Air or N_2_O at 50–70%
Animal position	• Prone position, fixed with tape or plastic pins • Nose cone for anesthesia • Breathing sensor around the abdomen • Temperature sensor by rectal probe
Hemodynamic parameters monitored	• Temperature normothermia at 35.5−37.5°C • Heart rate at 400−650 beats/min (strain dependent) • Breathing at 50−100 cycles/min
Prospective gating	• Several cardiac cycles, predefined cycle portion (e.g., diastole) measured simultaneously with ECG for synchronization
Echocardiography	• Multiphase gradient echocardiography-based cine cardiac imaging

While currently less widely available, more resource intensive, and with lower temporal resolution than echocardiography, advantages of using cardiac MRI in mouse models of heart failure include high accuracy and versatility (6, 167). Cardiac MRI in mice uses pulse sequences that not only allow assessment of volumes, mass, and systolic and diastolic physiology but also myocardial perfusion, flow, viability, and myocardial strain and anatomy.

### Cardiac Volumes, Mass, and Physiology

Cardiac MRI benefits from unrestricted spatial access to the myocardium, allowing long-axis imaging of two, three, or four chambers and continuous stacks of serial short-axis images for reproducible measurements of ventricular volumes and masses (9, 167, 184). Current instruments allow for a temporal resolution of 10–20 phases/heart beat (i.e., 5–10 ms) and a spatial resolution of 0.1–0.2 mm (188). A stack of six to eight serial, 1-mm-thick, short-axis slices is recommended to cover the entire LV and RV (184, 197).

Assessment of LV diastolic physiology by MRI in mice is more challenging. Nevertheless, there have been a few successful reports. For example, in a mouse model of dilated cardiomyopathy, Chłopicki and colleagues used images of the midventricular short-axis plane at the level of the papillary muscle to measure filling rates derived from time-area curves (44, 180). Other laboratories have also successfully used the same approach at higher fields of 7–9.4 T (1, 14, 196).

### Myocardial Perfusion and Blood Flow

MRI is a powerful tool for the assessment of myocardial perfusion (i.e., myocardial blood flow per gram of tissue, in ml·g^−1^·min^−1^), providing a means to characterize the relationship between blood flow oxygen delivery and cardiac contraction. There are currently two cardiac MRI approaches to evaluate myocardial perfusion: *1*) Gd-enhanced, first-pass perfusion after an intravenous bolus injection of an exogen contrast agent and *2*) arterial spin labeling using the intrinsic properties of water protons in blood as an endogenous tracer not requiring contrast injections (95). The Epstein laboratory (129) is a leader in the MRI field and has compared both techniques in mice. During low myocardial blood flow conditions, such as post-MI, the use of first-pass perfusion MRI is preferred, due to better reproducibility and lower variability. At high myocardial blood flow during vasodilation, arterial spin labeling may be more suitable, due to superior image quality and lower user variability. First-pass perfusion MRI has a substantial speed advantage and has successfully been applied in mouse MI models (34, 178), cardiac hypertrophy (186), and obesity (128, 130).

### Myocardial Viability

Similar to echocardiography, MRI can be used noninvasively to assess changes serially in the same mouse over time. One advantage for MRI is the ability to assess nonviable versus stunned myocardium and, therefore, to measure infarct area over time. This is achieved by late Gd-enhancement imaging after injection of a contrast agent (92). The technique is based on the principle that Gd chelates have an extravascular distribution volume (188). Late Gd enhancement images are acquired at least 10 min after an intravenous or intraperitoneal injection of the contrast agent. Infarcted or fibrotic myocardium presents decreased cellular volume and increased extracellular volume, which result in higher contrast concentrations at equilibrium, translating to shorter LV longitudinal relaxation times (T1) (81). Gd-based contrast dyes are low-molecular-weight extracellular agents that are small enough to move across the vascular wall into the extracellular space yet are large enough that they do not infiltrate cells with intact membranes (137). To obtain optimal T1 time between normal and ischemic myocardium, postcontrast imaging is performed with inversion recovery to subtract the signal intensity for normal myocardium with a look-locker sequence (138). For late Gd enhancement imaging in mice, field strengths of 4.7 T (176), 7 T (141), or 9.4 T are typically used (23), with segmented gradient-recalled echocardiography or fast low-angle shot sequences, both using multiechocardiographic acquisition. In 2011, Price et al. (138) proposed a faster late Gd enhancement imaging protocol using multislice acquisition that allows for higher flip angles and, therefore, higher signal-to-noise rate efficiency. This technique has been used for imaging of cardiac ischemia (131) and coronary artery plaques (77). Data can be confounded, however, in the presence of pathologies, where increased water is a symptom, such as edema and amyloidosis, which can be detected by an increase in transverse relaxation (T2 signal) (137). In these cases, cardiac T1 mapping without the use of a Gd-based contrast agent has been shown to be a sensitive approach in both human and mouse models (51, 89, 101, 174, 181).

### Myocardial Strain Mapping

Cardiovascular magnetic resonance tagging is an established technique for measuring regional myocardial function. It allows quantification of myocardial motion measures, such as strain and strain rate, by visualizing transmural myocardial movement without having to insert physical markers (73). This technique opened the door for a series of developments and technical improvements that continue to develop. Methods used for myocardial strain mapping in mice include the following: displacement encoding via stimulated echoes (DENSE), spatial modulation of magnetization (SPAMM)-tagged imaging, and harmonic phase (HARP) MRI. DENSE has the ability to extract myocardial motion data at high spatial density over segments of the cardiac cycle (4). DENSE MRI is particularly useful in the quantification of LV volumes and mass in mice (60) and to quantify cardiac displacement directly (56). In SPAMM-tagged MRI, the magnetization is modulated using radiofrequency pulses and magnetic field gradients (120). This results in saturated bands in the magnetization distribution and, as a result, contrasting patterns in the image data. Tissue tagging by SPAMM has often been used to access myocardial strain in humans and in animal models (37, 43, 72, 208). Zhou and colleagues (211) used SPAMM MRI in combination with a cine protocol to map myocardial strains and displacements in mice. HARP MRI allows for automated and fast analysis of high-tagging resolution images in mice (64, 104, 209, 210). HARP MRI has also proven useful to compare cardiac displacement and strain between cardiomyocyte-specific genotypes and wild-type mice (32). More recently, the introduction and coupling of the feature-tracking technique with MRI have allowed for the assessment of 3-D regional and global (longitudinal, circumferential, and radial strains) myocardial dysfunction (110, 194). While FT MRI has become a popular choice in the clinic for assessment of myocardial strain (12, 111), it is still in its infancy in animal models. Nonetheless, a recent study (99) evaluated FT MRI reproducibility in mice and reported good to excellent inter- and intraobserver reproducibility, suggesting that FT MRI shows analytic potential for experimental cardiac research. For a more complete list of myocardial strain-mapping techniques, see Ibrahim (73) and Jiang and Yu (82).

### Myocardial Anatomy

Cardiac muscle architecture directly correlates to the mechanical and electrical properties of the myocardium, and changes in fiber structure and orientation are of prime importance in post-MI remodeling assessment (149). Diffusion-encoded or diffusion-tensor MRI can be used to examine myocardial fiber orientation. Microstructural myocardial imaging, at the scale of individual myofiber tracts and sheets, has the potential to provide a mechanistic bridge between cellular and molecular events and the whole organ physiology (71). While in vivo cardiac diffusion tensor MRI is still in the early stages of development, ex vivo microscopic structural imaging is commonly used to assess myocardial structural remodeling after MI or aging (31, 103, 166, 200).

While cardiac MRI is a powerful, noninvasive, and versatile technique that provides high-quality cardiac images and allows for reproducible study of cardiac anatomy and physiology, there are some limitations. Major limitations with the use of MRI include cost (instrument and contrast agents), longer and more resource-intensive protocols, lower temporal resolution, signal-to-noise ratio limitations, and reduced availability (see Table 6 for a detailed list of advantages and limitations of MRI). While many clinical studies have compared data obtained from both echocardiography and MRI in the same patients (93, 94), experimental models comparing both modalities are limited. Experimental mouse models comparing the use of echocardiography with MRI noted good correlation in measures of volume and physiology (108). With the comparison of changes in cardiac structure, MRI was better at detecting moderate to severe diffuse myocardial fibrosis. A study by Li et al. (105) reported good correlation between radial strain and circumferential strain between 2-D echo and MRI short-axis views. Thus, echocardiography and MRI deliver comparable cardiac physiological measurements, with MRI offering increased versatility and potential benefit on studies focused on myocardial fibrosis and assessment of tissue viability.

**Table 6.** Recommended methods for cardiac physiological measurements: advantages and limitations of each methodology

Technique	Advantages	Limitations
Echocardiography	• High availability • Portable • Cheap • Available for individual laboratory use • Fast measurements (with experience) • Serial measurements • Simultaneous measurement of a wide range of physiological parameters • Allows assessment of chambers, pericardium, valves, strain, and function • Highest temporal resolution • Use of awake or anesthetized animals Awake: no effects of anesthesia Anesthetized: easier to handle mice and change probe location	• Technical variability (probe location, chamber trace) if operator is not highly trained • Need to quality control data acquisition and analysis • Acclimation needed, particularly for serial measurements in the same mouse • Awake Could cause stress. Heart rates of >650 beats/min generally reflect a stressed state Enrichment and training needed • Anesthetized Over-/underanesthesia: heart rates should be maintained at >400 to <650 beats/min to ensure physiological relevance
Cardiac MRI	• High accuracy and reproducibility • Versatile: allows assessment of chambers, pericardium, valves, strain, function, tissue viability, and perfusion • High spatial resolution • High tissue/blood contrast • Serial measurements	• Low availability • Not portable • Expensive • Uses contrast agents • Longer times necessary for measurements • Easy-to-introduce motion artifacts • Signal-to-noise ratio limitations • Cardiac gating necessary • Lower temporal resolution
Hemodynamics	• Allows pressure and volume assessments (depending on catheter) • Provides load-independent measures (e.g., end-systolic pressure-volume relationship)	• Technically challenging, need to quality control data acquisition and analysis • Nonsurvival procedure • Heart rates should be maintained at >400 beats/min, and mean blood pressure should be >90 mmHg to ensure physiological relevance

## HEMODYNAMICS

**Fig. 2.**
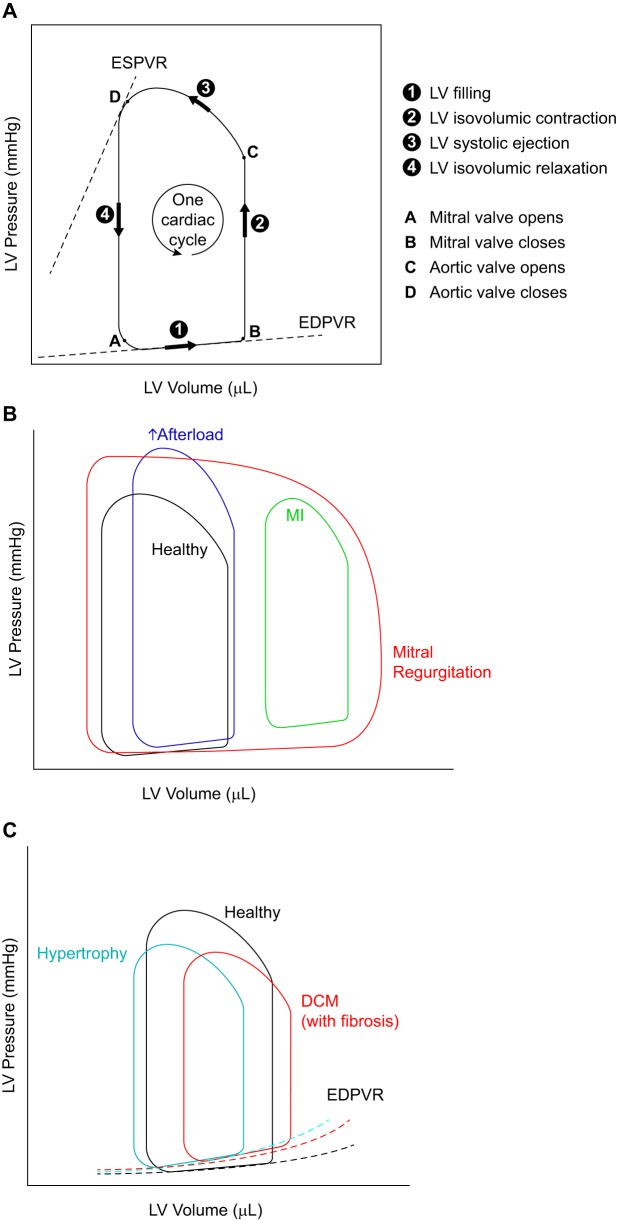
Illustrations of pressure-volume (P-V) loops and how they are altered in heart disease. *A*: P-V loops representing the changes in pressure and volume of the left ventricle (LV) during one cardiac cycle. Information in LV pressure and volume during different phases of a cardiac cycle can be obtained from this loop as indicated. *B*: the shape and relative location of the P-V loop are affected differently in various types of heart diseases. In mitral regurgitation, the width of the P-V loop does not represent the stroke volume, because not all of the blood is pumped out of the LV, due to the regurgitant mitral valve. The mitral regurgitation is also responsible for the absence of a true isovolumic relaxation or contraction. *C*: end-diastolic P-V relationship (EDPVR) can serve as a measure of myocardial stiffness (of the LV). A steeper slope for this curve correlates with increased stiffness (reduced compliance) of the LV myocardium. ESPVR, end-systolic pressure-volume relationship; DCM, dilated cardiomyopathy.

Invasive hemodynamic measurements can refine the information on cardiac physiology provided by echocardiography. This approach is not routinely used in mice as a firstline method, however, because this approach is technically challenging and is a nonsurvival procedure precluding this from being a serial assessment, and the results can be difficult to interpret. A micromanometer-tip catheter is inserted into the LV chamber (through the carotid artery, retrogradely or through the apex), where it can directly measure the changes in pressure (and volume when a conductance catheter is used) of the LV over time. Illustrations of pressure-volume loops and how they are altered in cardiac disease are shown in Fig. 2.

Parameters derived from LV pressure loops can be helpful, especially for the study of diastolic physiology. LV minimum pressure rates (dP/d*t*_min_) and the isovolumic relaxation constant (τ), although load dependent, are easier to interpret than echocardiographic mitral inflows in mice, which can be fused at high heart rates. RV pressure measurements are invaluable for a direct measure of pulmonary arterial systolic pressure. Pressure-volume loops are considered the gold standard for hemodynamic assessment of ventricular performance and are mostly obtained from the LV, although reports on RV performance have been made (169). The advantage of pressure-volume hemodynamic measurements over echocardiography is that some of the indexes, such as the end-systolic pressure-volume relationship (ESPVR or *E*_es_, described below), are considered to be relatively load independent (24, 134).

A number of pressure catheters and pressure-volume catheters are commercially available that range in size and sensitivity. A comparison of pressure catheters from Millar (Houston, TX), Scisense (Ithaca, NY), and RADI Medical Systems (Uppsala, Sweden) showed that pressure measurements by all catheters were stable, with a drift of ±2 mmHg within the 0- to 300-mmHg range. Of the three, the Millar amplifier had the shortest delay (0.2 ms) compared with Scisense (3.2 ms) and RADI Medical Systems (10.6 ms) amplifiers (63). For a heart rate of 500 beats/min in a mouse, a 10.6-ms delay translates to 8.3 beats/s, which should not impact recording frequency. The Scisense catheter can detect lateral forces with high sensitivity, which allows the catheter to register pressure from all surrounding regions rather than only the force exerted directly at the tip.

In addition, the Scisense catheter has the advantage of reporting the absolute LV volume without a need for calibration, while Millar pressure-volume catheters require volume calibration. This is achieved in the Scisense pressure-volume catheter, because when inserted into the LV, it measures the overall conductance as well as the parallel conductance coming from the surrounding myocardium (which changes during the cardiac cycle). Parallel conductance is automatically quantified and removed from the total conductance, thereby providing direct information on LV volume (blood volume in the LV chamber). With Millar pressure-volume catheters, the conductance catheter signal is proportional to volume (of blood in the LV) and must be appropriately calibrated to provide accurate absolute volume measurements. It must be emphasized that the calibration is a crucial and often technically difficult step in the use of conductance catheters. One step that we would suggest, in addition to the calibration proposed by the manufacturers (calibration using cuvettes of different sizes), is to validate the conductance-derived volumes using the echocardiography-derived volumes.

### Anesthesia and LV Catheterization

Similar to echocardiographic imaging, choice of anesthesia is important for reliable and reproducible assessment of hemodynamics. Isoflurane has become the anesthetic of choice in hemodynamic measurements in mice because of its minimal cardiosuppressant effects compared with ketamine-xylazine and pentobarbital sodium (80), although ketamine-fentanyl has been reported also to lack cardiosupressant effects (74). As mentioned earlier, an additional advantage of an inhalant anesthetic (isoflurane) over injectable options is that its levels can be monitored, and, therefore, the depth of anesthesia can be controlled during the procedure.

LV catheterization can be performed by a closed- or open-chest approach. In the open-chest approach, the catheter is inserted into the LV (or RV) through the apex, whereas in the closed-chest approach, the catheter is inserted in the carotid artery and extended into the ascending aorta and LV. The closed-chest approach has a number of advantages: arterial blood pressure can be recorded from the carotid at the beginning or end of the procedure, intubation is not required, and animals can remain stable for a longer time, which is ideal for prolonged procedures, such as drug testing. This approach is also preferred for hemodynamic measurements post-MI, since the LV infarction often extends to the apex, and insertion of the catheter through the scarred myocardium can be problematic. Open-chest LV catheterization also has advantages, since proper placement of the catheter in the LV is easier to confirm and is a better approach if the carotid artery is severely atherosclerotic (e.g., high-fat diet-fed apolipoprotein E or LDL receptor-deficient mice) or in cases of aortic valve calcification. In all cases, we would recommend the use of the 1-Fr catheter to ease the introduction of the catheter in the LV and to decrease the risk of obstruction of the LV cavity around the catheter with elevated and inaccurate systolic pressure recordings.

### Hemodynamic Data Analysis and Interpretation

dP/d*t*_max_ and dP/d*t*_min_ rise and decline can be derived from LV pressure traces (first derivative). LV τ can be calculated by the Weiss method, expressed as the regression of the pressure versus time logarithm, or by the Glantz method, expressed as the regression of dP/d*t* versus pressure (204). τ increases as LV relaxation decreases (115). Systolic dysfunction is noted by a decrease in dP/d*t*_max_, and diastolic dysfunction is detected by an increase in LV end-diastolic pressure and τ and a decrease in dP/d*t*_min_. Due to the small values of some parameters (e.g., LV end-diastolic pressure), calibration is crucial to minimize error. All parameters are load dependent.

With the use of a conductance catheter, parallel changes in LV pressure and volume can be recorded over consecutive cardiac cycles. A number of parameters can be obtained from the conductance catheters, including SV (SV = EDV – ESV), EF (EF = SV/EDV), CO (SV × heart rate), and arterial elastance (arterial elastance = end-systolic pressure/SV), where EDV is end-diastolic volume and ESV is end-systolic volume. Volume measurements rely on geometrica assumptions, which can make results difficult to interpret in asymmetrical volumes and for RV measurements (134). The pressure-volume relationship is presented as loops along the *y*-axis (pressure) and *x*-axis (volume).

In addition, with the adjustment of preload, i.e., by temporary occlusion and slow release of the inferior vena cava, the ESPVR and end-diastolic pressure-volume relationship (EDPVR) can be determined. ESPVR represents the maximal pressure developed by the LV at any given volume and is a measure of cardiac contractility. The slope of ESPVR, also referred to as *E*_es_, is an index of end-systolic elastance, an index that provides information on contractile function (134). The degree to which *E*_es_ is load dependent is under discussion (3). Ventriculoarterial coupling, which reflects the interaction of the heart and its afterload, can also be assessed using the ratio of *E*_es_/arterial elastance. Regarding the diastolic properties of the heart, EDPVR represents the passive filling properties of the LV, and the slope of this curve is a measure of myocardial compliance (reverse of stiffness). An increase in this slope will indicate increased myocardial stiffness or decreased compliance (162). Both *E*_es_ and EDPVR are dependent on chamber size and remodeling.

In hypertrophic cardiomyopathy, the LV walls become thicker and LV chamber size decreases or remains unchanged. The increased LV wall thickness can reduce compliance, resulting in an increase in EDPVR slope. During compensatory hypertrophy when systolic pressure is not suppressed, no change in end-systolic pressure (*y*-axis) will be observed. In dilated cardiomyopathy, ESV and EDV increase, but LV pressure can remain unchanged; therefore, the ESPVR and EDPVR are shifted to the right. If dilated cardiomyopathy is associated with fibrosis and diastolic dysfunction, then an upward shift in the EDPVR will be observed. After MI or other instances of volume overload, ESV and EDV increase, end-systolic pressure decreases, and end-diastolic pressure increases; as a result, the pressure-volume loop will look smaller (shorter) with a rightward shift (see Fig. 2).

## OVERALL DISCUSSION AND CONCLUSIONS

As highlighted throughout these guidelines, the measurement of cardiac physiology is a critical component of cardiovascular research. Methods for the accomplishment of this include echocardiography, MRI, and hemodynamic evaluation using pressure-volume catheters. A summary of overall recommendations, including strengths and limitations of each technique, is shown in Table 6. The approach used will vary depending on the questions being addressed; as such, all of the approaches described above may be considered an appropriate approach if they answer the target hypothesis. While echocardiography is the most frequently used technique, due to its availability, technical ease of use, capacity for serial imaging, and cost−all methods discussed in this guidelines article−provide excellent means to evaluate cardiac physiology and can be complementary to each other. A combination of methods is frequently used to overlap limitations of one approach with strengths of the other (10).

**Fig. 3.**
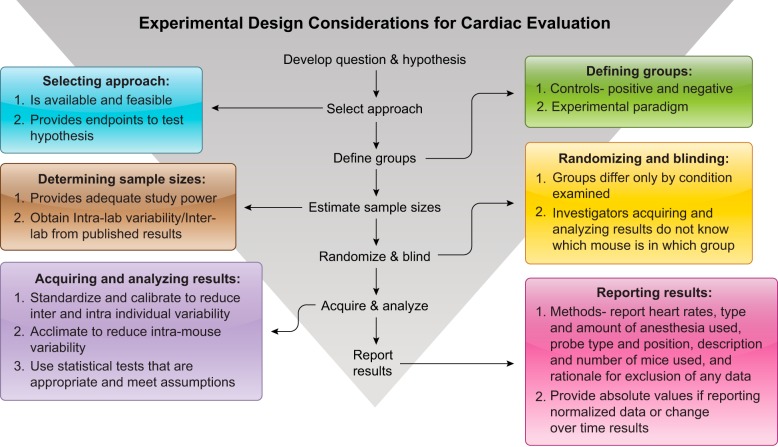
Experimental design considerations for studies measuring cardiac physiology indexes.

Prospective planning of study design (i.e., randomization for appropriate control vs. treatment, blinding, and adequate statistics) is mandatory for reproducibility of all experimental approaches, and cardiac physiology experiments are no exception (Fig. 3). The absolute values of the results and the components entering into a calculation should be reported for any normalized measurements. For example, studies that only report changes in calculated measurements, such as EF, do not enable the field to know whether the altered EF resulted from a change in systolic volume, diastolic volume, or both, and this lack of details confounds our ability to understand the underlying mechanisms of altered physiology. Additionally, there are an increasing number of computational models and tools being developed to understand cardiac physiology, predict disease-related remodeling, and design novel interventional therapies. The accuracy of these models are dependent on comprehensive measurements of cardiac dimensions and function under various circumstances. Reporting only percent change without showing absolute values forces model developers to make assumptions for the missing data.

The ultimate validation is confirmation of results across individuals and across laboratories. As larger data sets are being acquired, consideration for how to harness big data and evaluate cardiac physiology results across laboratories should be given (156, 157). This will be a particular challenge, as values obtained from imaging approaches can be affected by both acquisition and analysis conditions. The compilation of databases to incorporate results from across studies and across laboratories will provide a means to use epidemiological approaches or big data tools to validate published findings, generate novel hypotheses, and assess individual variability in cardiac structure and function. In conclusion, these guidelines provide recommendations to help the investigator plan and execute a full range of studies involving cardiac physiology.

## GRANTS

Support from the following funding agencies is acknowledged by the authors: National Heart, Lung, and Blood Institute Grants HL-075360, HL-129823, HL-051971, and HL-131613; National Institute of General Medical Science Grants GM-104357 and GM-114833; American Heart Association Grant 14SDG18860050; Biomedical Laboratory Research and Development Service of the Veterans Affairs Office of Research and Development Grant 5I01BX000505; Heart and Stroke Foundation (Canada); and Canadian Institute of Health Research.

## DISCLOSURES

The content is solely the responsibility of the authors and does not necessarily represent the official views of any of the funding agencies listed. No conflicts of interest, financial or otherwise, are declared by the authors.

## AUTHOR CONTRIBUTIONS

M.L.L. conceived and designed research; M.L.L. prepared figures; M.L.L., Z.K., J.A.I.V., L.E.d.C.B., and M.S-C. drafted manuscript; M.L.L., Z.K., J.A.I.V., L.E.d.C.B., and M.S-C. edited and revised manuscript; M.L.L., Z.K., J.A.I.V., L.E.d.C.B., and M.S-C. approved final version of manuscript.
